# Supplementary material for: RiboTRAP-seq identifies spatially distinct functions for the anterior and posterior intestine in immune and metabolic regulation in Caenorhabditis elegans
Source: eLife. 2026 Oct 16;15:RP109237. doi: 10.7554/eLife.109237 (PMC13675006; doi:10.7554/eLife.109237)
Supplement: Supplementary file 2 [file elife-109237-supp8.docx]

**RiboTRAP Protocol**

Adapted from Flavell lab protocol

Bio-protocol 9(15): e3328.

DOI:10.21769/BioProtoc.3328

**Grow age-synchronized animals to the appropriate developmental stage (2-8 eggs)**

1. Pour 15 cm enriched-peptone NGM plates and allow them to dry at room temperature overnight.
2. These plates allow for robust bacterial growth that can support a very dense nematode culture.
3. Seed enriched-peptone NGM plates (one per condition) with 1 mL OP50 and allow to dry. *Don’t spread OP50 too much over plate & dry with fume hood closed
4. Pick 125 young adult animals onto each plate and allow them to grow for 3 days @ 20 °C, until there are many adult progeny and eggs on the plate. Each plate will give you 75,000 – 100,000 worms from an egg prep.
5. Egg prep worms to bleach-synchronize progeny, rotate overnight in M9 buffer.
6. Eggs obtained from the above procedure can be plated directly on a freshly-seeded enriched-peptone plate. Each plate can support ~50,000 worms grown up to adult stage.
7. Allow worms to grow to adult stage ~74 hours at 20 °C with a 15 °C O/N step the day after plating. *Worm stage can be slightly varied, but the majority should be day 1 adults (2-8 eggs)

**Wash, freeze, and lyse animals in the presence of cycloheximide**

*At least one day in advance, prepare liquid NGM, homogenization buffer stock, and wash buffer stocks (see Recipes). Liquid NGM can be stored at room temperature, and homogenization and wash buffers are stable at 4 °C for one month.

*On the day of harvesting, add fresh reagents (DTT, protease inhibitor, RNasin, and VRC) to an aliquot of homogenization buffer (1 mL per plate).

*Prepare cycloheximide stock and add the stock to the following solutions at a final concentration of 0.8 mg/mL (125x dilution): liquid NGM (20 mL per plate), homogenization buffer (10 mL per plate), and homogenization buffer with fresh reagents (1 mL per plate).

*Clean your workstation, equipment, and gloves thoroughly with RNase ZAP.

*For starvation and refeeding, follow typical protocol using PBS to wash worms.

1. Wash worms off each plate in 10 mL liquid NGM with cycloheximide and transfer them into a 15 mL conical tube with a serological pipette. Take care not to disrupt the bacterial lawn during the wash to avoid transferring excess bacteria to the tube.
2. Wash 2x with 10 mL liquid NGM with cycloheximide. *Fasting groups only need to be washed once
3. Spin at 1,300 *x g* for 30 s, aspirate solution, then wash worms with 10 mL homogenization buffer with cycloheximide and transfer the solution to a fresh 15 mL conical tube.
4. Spin at 1,300 *x g* for 30 s, aspirate solution, and add 1 mL homogenization buffer with cycloheximide and fresh reagents.
5. Using an RNase-free pipette, drip the worm suspension into the 2 mL RNase-free tubes (round bottom) filled with liquid nitrogen, splitting the worm suspension evenly across five tubes. Place a small drop in a sixth tube that will be later used to estimate worm concentration. The worm suspension will bead up and freeze immediately upon contact with the liquid nitrogen. *Make sure liquid nitrogen is evaporated before closing cap
6. Store tubes at -80 °C.

**Estimation of worm concentration**

1. To estimate worm concentration, thaw the extra tube of frozen worms, then dilute it 10x and pipet 10 μL onto a glass slide.
2. Count the number of worms present in the droplet.
3. Repeat this 2x and calculate the average to estimate the number of worms per μL in each frozen sample.

**Grind the frozen worm pellets using the RNase-free pestles**

1. Add liquid nitrogen to an RNase-free ice bucket with a cardboard insert, as before. And fill a 50 mL RNase-free conical tube with liquid nitrogen to act as a reservoir.
2. Cool a pestle by dipping it into an RNase-free microcentrifuge tube filled with liquid nitrogen.
3. Remove 1 set of samples from the -80 °C freezer and place it in the bucket. Grind the sample thoroughly with a pestle, ensuring that it remains frozen and will be ground up into a fine powder. Whenever the sample begins to thaw slightly, cool it again by pouring liquid nitrogen over it. Move ground up samples to -80 °C

*Continually cool pestile

*Time consuming

*Grind carefully to avoid losing sample out of tube

4. Repeat for all tubes until all samples have been ground into powder and stored at -80 °C

*Prepare the liquid NGM, homogenization buffer stock, 150 mM KCl wash buffer stock, and 350 mM KCl wash buffer stock at least one day before performing the immunoprecipitation. The homogenization buffer and wash buffers can be stored for up to one month at 4 °C

**Perform immunoprecipitation**

*Clean your workstation and all equipment thoroughly with RNAse Zap. All of the following steps should be performed on ice, keeping the samples cold throughout.

1. Thaw the worm powder by diluting it into cold homogenization buffer (with all fresh ingredients
2. added) to a concentration of 8 worms/μL and transfer it to a 15 mL conical tube. Based on the total volume of extract used. Ensure that samples remain cold to prevent RNA degradation.
3. Use a small known volume of homogenization buffer to gently resuspend the worm powder in each microcentrifuge tube.
4. Once all powder is transferred to the 15 mL tube, measure the displacement with a serological pipette to determine the amount of powder added to the tube, then add extra homogenization buffer to bring the solution to the correct concentration. *This dilution is very important, high concentrations of animals can lead to substantial RNA degradation, presumably due to endogenous RNases.
5. Add 1/10 volume of 10% NP-40 and 1/9 volume of 300 mM DHPC to the 15 mL tube. Incubate on ice for 10 min, inverting occasionally.
6. Transfer the solution to RNase-free microcentrifuge tubes and spin for 12 min at 15,000 *x g* at 4 °C. Transfer the supernatant into fresh tubes, then centrifuge again, and pool the supernatant into a fresh 15 mL RNase-free conical tube.
7. While centrifuging the samples, wash the Protein G beads in 150 mM KCl Wash Buffer. Add 1 mL of 150 mM KCl Wash Buffer to 300 μL beads (per IP); use 150 μL beads for pre-clearing, collect the beads with a magnetic rack, aspirate wash buffer, and repeat 2 x.
8. To pre-clear the lysate, add 150 μL washed Protein G beads to the lysate and rotate at 4 °C for 30 min, then collect the beads with the magnetic rack and transfer the cleared supernatant to a fresh 15 mL conical tube.
9. Collect a 100 μL “input” sample at this point and mix with 100 μL of lysis buffer from the Agilent Absolutely RNA Nanoprep Kit. Ensure that beta-mercaptoethanol (BME) has been added to the lysis buffer, as per kit protocol. Vortex, let sit at room temperature for 10 min, vortex again, then store at -80 °C.
10. Add 15 μL monoclonal anti-HA antibody to pre-cleared lysate and rotate at 4 °C for 30 min.
11. Add 150 μL Protein G beads to the lysate and rotate at 4 °C for 30 min.
12. Place the 15 mL conical tube on the magnetic rack and collect a 100 μL “flowthrough” sample and process in the same manner as the “input” material. This sample is valuable to check if there was any RNA degradation during the incubation with antibody and beads.
13. Wash twice with 350 mM KCl Wash Buffer, each time gently inverting the tube then placing it on the magnetic rack to aspirate supernatant. Resuspend the beads in 350 mM KCl Wash Buffer and transfer to a clean RNase-free microcentrifuge tube. Wash once more with 350 mM KCl Wash Buffer, place on the magnetic rack, and aspirate supernatant (in total, the beads should be washed 4x with 350 mM KCl Wash Buffer).
14. Elute RNA by adding 125 μL lysis buffer with BME (from the Agilent Absolutely RNA Nanoprep Kit), vortex, and let the sample sit for 10 min at room temperature with occasional agitation. Place the tube on the magnetic rack, collect liquid in a fresh, chilled RNase-free microcentrifuge tube, and store at -80 °C. Beads can be discarded after elution.
