# Supplementary material for: RiboTRAP-seq identifies spatially distinct functions for the anterior and posterior intestine in immune and metabolic regulation in Caenorhabditis elegans
Source: eLife. 2026 Oct 16;15:RP109237. doi: 10.7554/eLife.109237 (PMC13675006; doi:10.7554/eLife.109237)
Supplement: Peer review history

# Editor report: eLife Assessment

**DOI:** 10.7554/eLife.109237.3.sa0

**Authors:** Sylvia Lee

**Affiliations:** Cornell University United States

**Keywords:** Solid, Valuable

In this **valuable** study, the authors performed cell-specific ribosome pulldown to identify gene expression (translatome) differences in the anterior (NT1) vs. middle and posterior (NT2-9) cells of the *C. elegans* intestine, underfed, starved, or refeeding conditions. The data generated will be very helpful to the *C. elegans* community, and the evidence supporting the conclusions of the study is assessed to be **solid**. Some methodological caveats remain and are discussed.

# Referee report: Reviewer #1 (Public review)

**DOI:** 10.7554/eLife.109237.3.sa1

Summary

In this study, the authors have performed tissue-specific ribosome pulldown to identify gene expression (translatome) differences in the anterior vs posterior cells of the *C. elegans* intestine. They have performed this analysis in fed and fasted states of the animal. The data generated will be very useful to the *C. elegans* community, and the role of pyruvate shown in this study will result in interesting follow-up investigations.

However, several strong claims made in the study are solely based on in silico predictions and are not supported by experimental evidence.

Comments on revised version.

The authors have been responsive to the comments, but have not added new experiments in this manuscript that would have clarified and improved some of the mentioned shortcomings of the study.

There are 3 comments that the authors should address:

(1) In their response to reviewers, the authors agree that "the Pges-1deltaB promoter is not absolutely restricted to INT1 and that weak GFP expression can also be detected in INT2." They also mention that "because Pges-1deltaB is an engineered promoter derived from the intestine-specific Pges-1 promoter, this low-level INT2 expression is not unexpected." However, in line 93 of the revised manuscript, the authors claim that "Pges-1deltaB is strictly expressed in INT1 cells". This discrepancy should be fixed. They should instead describe this in line 93 as "Pges-1deltaB expression is very strongly enriched in INT1 cells, but low-level expression in INT2 was also detected".

(2) In response to reviewers, the authors explained that "Our model is that fasting induces INS-7 secretion by lowering intracellular pyruvate in INT1 cells. Under this framework, blocking mitochondrial pyruvate breakdown would be expected to reduce pyruvate utilization and thus maintain intracellular pyruvate, preventing the drop in pyruvate that normally occurs during fasting. This would explain why these manipulations suppress fasting-induced INS-7 secretion." However, the effect of blocking import of pyruvate from cytosol into mitochondria (via knockdown of mitochondrial pyruvate carrier genes mpc-1 and mpc-2) does not agree with their proposed model. Blocking mitochondrial import of pyruvate should maintain cytosolic pyruvate levels and thus prevent the drop in pyruvate that normally occurs during fasting. In such a scenario, we would expect to see no increase in INS-7 secretion during fasting, which is opposite to the result in Fig.7D. If the pyruvate sensor is in the cytosol, we would expect that the mpc-1/2 RNAi treated animals would be unable to increase INS-7 secretion upon starvation. If the pyruvate sensor is in the mitochondrial matrix, we would expect that the mpc-1/2 RNAi treated animals would have higher INS-7 secretion than vector RNAi control animals in fed conditions. How do the authors explain this discrepancy between their observed results and their proposed model? Why does blocking mitochondrial import of pyruvate affect only refeeding-induced reduction in INS-7 secretion but not fasting-induced increase in INS-7 secretion? Is it possible that instead of responding to absolute intracellular concentrations of pyruvate, the pyruvate sensor increases INS-7 secretion upon detecting a relative drop in the mitochondrial levels of pyruvate (or its downstream metabolite)? This should be described in the text to better interpret the mpc-1/2 RNAi results.

(3) Line 493: The authors refer to 'Table S4', which is not included in the manuscript.

# Referee report: Reviewer #3 (Public review)

**DOI:** 10.7554/eLife.109237.3.sa2

In this study, Liu and colleagues utilize TRAP-seq to profile the repertoire of actively translated mRNAs in different intestinal cell types (anterior INT1 vs. posterior INT2-9 cells) in *C. elegans*. A key goal of this study was to identify transcripts differentially expressed/translated between these intestinal cell subtypes in the context of animals being well fed or subjected to acute (30 minutes) or chronic (3 hours) starvation, followed by refeeding.

The authors identify a number of differentially expressed genes across all of the conditions tested. They then provide an initial survey of the landscape of translatome changes through Weighted Gene Network Correlation Analysis (WGNA), and some high-level functional surveys via Gene Ontology (GO) term analysis and protein domain analysis. The authors validate the enriched expression patterns of some of their identified candidate genes using fluorescent promoter fusion reporters, confirming INT1-specific expression. The authors further implicate the role of several other candidate genes in pathogen avoidance and in response to nutritional cues by knocking them down specifically in INT1 cells by RNAi. Finally, the authors identify pyruvate as a major nutrient signal coming from the bacterial diet that suppresses the release of a key insulin peptide (INS-7) and identify some of the genes expressed in INT1 that are required for this response.

Strengths:

(1) Good use of and justification for TRAP-seq, because scRNA-seq would be difficult under the varied conditions used (starvation, refeeding)

(2) The manuscript is generally clear to read, and the data are generally well-presented with good supporting data that includes replicates, sample sizes, error measurements, and associated statistics.

(3) The dataset will be an interesting resource to mine for future studies focusing on mechanisms of how particular intestinal cell types respond to different environmental signals.

Weaknesses:

(1) A limitation of TRAP-seq, although powerful, is that only relative comparisons can be made between genotypes/conditions to identify differentially-expressed genes, rather than assessing whether a given gene is expressed at a certain level in a cell type under a certain condition. This limitation is due to the non-specific association of sticky RNA species to the beads during the immunoprecipitation step. This is a minor point however, and the authors do a nice job of focusing their analysis on differentially expressed transcripts in the current study.

(2) Another limitation of the current study is that the experiments testing the role of candidate genes identified by their profiling experiments do not dive a bit deeper into providing a mechanistic understanding of the phenotypes being studied. At present, the results are thus viewed more as a genomics-based screen with some limited follow-up on interesting hits. However, this reviewer appreciates that when placed in context of the work presented, a presentation of the profiling data along with some validation is an excellent starting point for future mechanistic studies elaborating on these interesting candidates.

Appraisal of whether the authors achieved their aims, and whether the results support their conclusions.

The main goal of the study was to survey the dynamic responses at the level of actively translated mRNAs of the INT1 vs INT2-9 cells in response to metabolic challenge.

Overall, the authors use established methods to perform their genome-wide analysis, and the set of differentially regulated genes are enriched for expected molecular functions and form coherent networks in anticipated pathways.

The validation experiments (promoter::GFP fusion reporters, INT1-specific knockdowns of highly regulated genes) further corroborate the quality of the TRAP-seq datasets generated.

I have a few points for the authors that would further strengthen this work:

(1) The authors rightfully focus on the top differentially-regulated candidates, but it's unclear at present how far down their fold change list would lead to expression pattern validations. It would be useful to test a few more promoter::GFP fusion reporters at different enrichment/fold-change/statistical cutoffs.

(2) Although the INT1-specific RNAi provides a convenient strategy for rapidly perturbing and testing genes of interest for phenotypes, independently validating the knockdowns with genetic mutants, or alternatively (if genes are essential), degron alleles.

Likely impact of the work on the field, and the utility of the methods and data to the community.

The TRAP-seq data and list of differentially-expressed candidate genes will form an interesting set of high-priority candidates to study for their role in the reception and transduction of nutritional cues in response to food status and pathogens. This data will thus benefit the *C. elegans* community of researchers studying the mechanisms governing these phenomena.

Comments on revised version:

I think the authors have done a good job of addressing the suggestions from the previous round of review in this new version.

# Author comment: Author response

**DOI:** 10.7554/eLife.109237.3.sa3

**Authors:** Chung-Chih Liu, Nicolas Seban, Supriya Srinivasan

**Affiliations:** Scripps Research Institute La Jolla United States; Department of Psychiatry and Behavioral Sciences, University of California Davis Davis United States; Scripps Research Institute La Jolla United States

The following is the authors’ response to the original reviews.

**Public Reviews:**

**Reviewer #1 (Public review):**

Summary

In this study, the authors have performed tissue-specific ribosome pulldown to identify gene expression (translatome) differences in the anterior vs posterior cells of the *C. elegans* intestine. They have performed this analysis in fed and fasted states of the animal. The data generated will be very useful to the *C. elegans* community, and the role of pyruvate shown in this study will result in interesting follow-up investigations.

However, several strong claims made in the study are solely based on in silico predictions and are not supported by experimental evidence.

Strengths:

Several studies in the past have predicted different functions of the anterior (INT1) vs posterior (INT2-9) epithelial cells of the *C. elegans* intestine based on their anatomy and ultrastructure, but detailed characterization of differences in gene expression between these cell types (and whether indeed these are different 'cell types') was lacking prior to this study. The genes and drivers identified to be exclusively expressed in the anterior vs posterior segments of the intestine will be very helpful to selectively modulate different parts of the *C. elegans* intestine in future studies.

Another strength of this study is the careful experimental design to test how the anterior vs posterior cell types of the intestine respond differently to food deprivation and recovery after return to food. These comparisons between 'states' of a cell in different physiological conditions are difficult to pick up in single-cell analyses due to low sequencing depth, which can fail to identify subtle modulation of gene expression.

The TRAP-associated bulk RNA-seq approach used in this study is more suitable for such comparisons and provides additional information on post-transcriptional regulation during metabolic stress.

A key finding of this study is that pyruvate levels modulate the translation state of anterior intestinal cells during fasting. Characterization of pyruvate metabolism genes, especially of the enzymes involved in its mitochondrial breakdown, provides novel insights into how gut epithelial cells respond to the acute absence of food.

Weaknesses:

Unlike previous TRAP-seq studies (PMID: 30580965, 36044259, 36977417) that reported sequencing data for both input and IP samples, this study only reports the sequencing data for IP samples. Since biochemical pulldowns are variable across replicates, it is difficult to know if the observed differences between different conditions are due to biological factors or differences in IP efficiency. More importantly, since two different TRAP lines were utilized in this study and a large proportion of the results focus on the differences between the translational profiles of INT1 vs INT2-9 cells, it is essential to know if the IP worked with similar efficiency for both TRAP strains that likely have different expression levels of the HA-tagged ribosomal protein. One way to estimate this would be to perform qRT-PCR of genes that are known to be enriched in all intestinal cells and determine whether their fold-enrichment over housekeeping genes (normalized to input) is similar in INT1 vs INT2-9 TRAP strains and across the fed vs fasted conditions. The authors, in fact, mention variability across biological replicates, due to which certain replicates were excluded from their WGCNA analysis.

We appreciate the reviewer's comments. We agree that the lack of matched input sequencing libraries limits our ability to directly assess IP efficiency across replicates, conditions, and TRAP strains. However, several features of the dataset support the conclusion that the major differences reported here reflect biological rather than purely technical variation. First, the RPL-22-3xHA construct was integrated into each line to improve consistency across experiments. Second, although the INT2-9 TRAP strain yielded more RNA than the INT1 strain, as expected given the larger number of labeled cells, downstream analyses were performed on normalized count data rather than raw counts. Third, principal component analysis showed robust separation by promoter identity across all conditions, and expected INT1-enriched genes such as *ins-7* were recovered in the INT1 dataset. Together, these observations support the interpretation that the TRAP datasets capture reproducible, cell-type-specific differences in ribosome-associated transcripts. Nonetheless, we agree that direct input-normalized measurements would further strengthen the study, and we will explicitly note this as an important limitation.

It appears that GFP expression is also detectable in INT2 (in addition to strong expression in INT1 in Fig.1A). Compared to INT3-9, which looks red, INT2 cells appear yellow, suggesting that the expression patterns of the two TRAP drivers are not mutually exclusive, which changes the interpretation of many of the results described in the study.

We agree that the *Pges-1ΔB* promoter is not absolutely restricted to INT1 and that weak GFP expression can also be detected in INT2. Because *Pges-1ΔB* is an engineered promoter derived from the intestine-specific *Pges-11* promoter, this low-level INT2 expression is not unexpected. However, we note that the expression level in INT1 is substantially higher than in INT2. Thus, although the expression patterns of the two TRAP drivers are not completely mutually exclusive, *Pges-1ΔB* still provides the most selective available tool for enriching the INT1 translatome in the context of the current study.

Some parts of the study overemphasize the differences between the INT1 vs INT2-9 cell types, which is a biased representation of the results. For example, the authors specifically point out that 270 genes are differentially expressed in opposite directions in INT1 vs INT2-9 cell types during acute (30 min) fasting without mentioning the 1,268 genes that are differentially expressed in the same direction. They also do not mention here that 96% of the genes are differentially expressed in the same direction in INT1 and INT2-9 cell types after prolonged (180 min) fasting, suggesting that the divergent translational responses of these cell types are only observed in the first 30 minutes of food deprivation. Similar results have also been reported for the effect of fasting on locomotory and feeding behaviors, where 30 min of fasting produces more variable effects, which become more consistent after longer periods of fasting (PMID: 36083280). Hence, the effects of brief food deprivation should be interpreted with caution.

The intestine functions as a discrete and cohesive organ, so the expected result is that there would be no differences across the different cell types. For us, the surprise was that, in fact, there are differences between these cells at all. However, the point is well taken, and we have added a statement in the text to reflect that many genes change similarly in INT1 and INT2-9, while the differences reflect important functional divergence between these cell types.

Many of the interpretations of this study primarily rely on pathway enrichment analyses, which are based on the known function of genes. The function of uncharacterized genes that were found to be differentially expressed in INT1 vs INT2-9 cell types, e.g., the ShKT proteins, was not explored in this study. In addition, overreliance on pathway enrichment tools (instead of functional validation) has resulted in several conflicting findings. For example, one of the main messages of this study is that INT1 cells specialize in immune and stress response in response to fasting, which relies on pathway analysis in Figs 5E and 5F. However, pathway analysis at a different time point (shown in Figure S5A) indicates that INT2-9 cells show a much stronger increase in translation of stress and pathogen-responsive genes compared to INT1 cells. Hence, some of the results should be interpreted as different translational effects in INT1 vs INT2-9 cells after different lengths of food deprivation, without making broad claims about selective pathways being affected only in specific cell types.

We agree that some interpretations in the manuscript relied heavily on pathway enrichment analyses and should be stated more cautiously. In particular, we agree that the current data are most consistent with state-dependent differences in translational responses between INT1 and INT2-9 cells across different durations of food deprivation, rather than with the strongest version of a claim that specific pathways are selectively engaged only in one intestinal subset. We also agree that uncharacterized genes, including the ShKT family, were not mechanistically explored in the present study and should be presented as important candidates for future investigation.

The authors have compared their TRAP-seq results with genes enriched in the anterior and posterior intestine clusters from a previously published whole-animal adult scRNA dataset (PMID: 37352352). They claim that their TRAP-seq results are in agreement with the findings of the scRNA study. However, among the 10 genes from the 'posterior intestine' scRNA cluster in Fig.S1E, six are downregulated in the INT1 vs INT2-9 comparison, while four are upregulated. Hence, there is no clear agreement between the two studies in terms of the top enriched genes in the anterior vs posterior intestine, which should be considered for cross-study comparisons in the future.

We have removed the original Figure S1C–E, replacing it with a more informative analysis. The genes in the original panel were drawn from the top markers reported for intestinal clusters in Ghaddar et al. (PMID: 37352352). However, these markers were defined by comparison with all *C. elegans* cell types, rather than by comparisons among anterior, middle, and posterior intestinal populations, and are therefore not optimal for resolving differences between intestinal subregions. We instead assessed the expression levels of our INT1 up-regulated genes in their intestinal cluster and found that they have higher expression in the anterior intestine cluster (new Figure S1C). These results underscore the strength of our dataset for identifying genes that distinguish INT1 from INT2–9.

The authors describe in the manuscript that they have performed INT1-specific RNAi for two C-type lectin genes that are upregulated during fasting. Due to a recent expansion of C-type lectin genes in *C. elegans*, there is a high chance of off-target effects of RNAi that is designed for members of this gene family. More trustworthy results could have been obtained using CRISPR-based loss-of-function alleles for these genes, one of which is publicly available. Also, the authors do not provide any explanation for why knockdown of these stress-response genes, which are activated in INT1 cells in response to food deprivation, results in improved resistance to pathogens. This, in fact, suggests a role of INT1 cells in increasing pathogen susceptibility, and not pathogen resistance, during food deprivation.

We agree that RNAi targeting C-type lectin family members may be susceptible to off-target effects, and that validation with CRISPR null alleles, where available, would strengthen these findings. In the current study, we used INT1-specific RNAi as a cell-specific first-pass approach to test candidate gene function. We also agree that the pathogen phenotype requires cautious interpretation. Specifically, the finding that knockdown of fasting-induced INT1 lectin genes improves pathogen resistance does not support a simple protective model for these genes. Instead, it suggests that INT1-expressed stress-response genes modulate host susceptibility or host-pathogen interactions.

Many of the studies in this field (e.g., references 2-4 in this article) have investigated the effects of food deprivation ranging from 4 hr to 24 hr, which results in activation of starvation responses in *C. elegans*. In contrast, the authors have used shorter time periods of fasting (30 min and 180 min), and most of their follow-up experiments have used 30 min of food deprivation. Previous work has shown that the effects of food deprivation can either accumulate over time (i.e., the effect gets stronger with longer food deprivation) or can be transient (i.e., only observed briefly after removal of food and not observed during long-term food deprivation). Starvation-induced transcription factors such as DAF-16/FoxO and HLH-30 show strong translocation to the nucleus only after 30 min of fasting. Though gene expression changes in all stages of food deprivation are of biological relevance, the authors have missed the opportunity to explore whether increased INS-7 secretion from the anterior intestine is dependent on these starvation-induced transcription factors (which can be easily tested using loss-of-function alleles) or is due to other fast-acting regulatory mechanisms induced due to the absence of food contents in the gut lumen. A previous study (PMID: 40991693) has shown that DAF-16 activation during prolonged starvation shuts down insulin peptide secretion from the intestinal epithelial cells. Hence, it is not clear if increased INS-7 secretion is only a feature of short-term food deprivation or is also a signature of long-term starvation (e.g., at 8 hr or 16 hr timepoints). Since most of the INS-7 secretion data in this study are for 30 min of fasting, it remains unknown whether the discovered regulators of INS-7 secretion can be generalized for extended food deprivation that triggers major metabolic changes, such as fat loss (e.g., conditions shown in Figure 1D).

We agree that short-term food deprivation and prolonged starvation likely engage distinct regulatory mechanisms, and that our study primarily addresses an early phase of food deprivation rather than the full spectrum of starvation responses described in prior work. We selected the 30 min fasting condition because our previous study showed that INS-7 secretion is induced within this interval and returns to baseline upon refeeding, even before detectable intestinal fat loss. We also included a 180 min fasting condition to capture a later state associated with metabolic changes. However, we agree that the present study does not determine whether the regulators of INS-7 secretion identified here also govern secretion during more prolonged starvation (for example, 8 hr or 16 hr), nor does it test whether starvation-responsive transcription factors such as DAF-16 or HLH-30 contribute to this regulation. We appreciate that determining how this response transitions during prolonged starvation will be an important direction for future work.

Two previous studies (PMID: 18025456, 40991693) have shown a strong reduction in the expression of ins-7 in the anterior intestine using GFP-based reporters (both promoter fusions and endogenous CRISPR-generated) and in whole-animal RNA-seq data from starved animals. These results are in contrast to the increased INS-7 secretion from INT1 cells during fasting that is reported in this study. The authors here have reported that INS-7 translation is higher in INT1 compared to INT2-9 during fed, acute fasted, and chronic fasted conditions, but they have not shown whether INS-7 translation is upregulated during acute and chronic fasting in INT1 cells in their TRAP-seq analysis. Knowing whether increased INS-7 secretion during acute fasting is due to increased transcription, translation, or secretion of INS-7 is crucial to resolve the discrepancy between these studies.

In our dataset, INS-7 translation in INT1 tended to increase during acute fasting relative to the fed state (log_2_FC = 0.69), although this effect did not reach statistical significance after adjustment for multiple comparisons. Consistent with this trend, our secretion assay showed that INS-7 release from INT1 increases during fasting. However, we agree that the current data do not distinguish whether this increase in secretion is driven by enhanced synthesis, regulated release of pre-existing peptide stores, or a combination of both.

**Reviewer #2 (Public review):**

Summary:

In this study, the authors set out to understand whether the discrete segments of the *C. elegans* intestine were specialized to carry out distinct functions during an animal's exposure and adaptation to a fast-changing nutrient environment. To achieve this, the authors used a method called Translating ribosome affinity purification (TRAP), which provides a snapshot of what genes are being translated into proteins (and therefore functionally prioritized by the animal) under different fasting and re-feeding conditions. By expressing the TRAP constructs in two distinct segments of the intestine (INT1) and (INT2-9), the authors were able to identify how these segments responded to changing nutrient availability.

Already under steady state nutrient conditions, the authors found that INT1 and INT2-9 appeared to have different 'tasks', with INT1 expressing more immune- and stress-response related genes. Exposing animals to different regimens of starvation and refeeding also showed marked differences between the intestinal segments, and the gene expression patterns in INT1 were consistent with INT1 cells playing an integrative role in linking nutrient cues to the secretion of insulin molecules that regulate fat metabolism with food intake. In summary, the data presented catalogue, for the first time, gene expression differences between two areas of the intestine, suspected to play different roles, and through clever experiments, links these gene expression changes to responses to nutrient availability.

Strengths:

The data presented catalogue - for the first time and in a careful manner - gene expression differences between two areas of the intestine. They strongly support the presence of intriguing differences between two areas of the intestine in immune, metabolic, and stress-response regulation, and link these gene expression changes to the responses of these regions to nutrient availability.

Weaknesses:

The conclusions of this paper are mostly well-supported by data, but the relevance of the changing gene expression patterns could be better clarified and extended in the discussion.

We thank the reviewer for this constructive comment. In the revised manuscript, we have now expanded the discussion to more clearly interpret these dynamic translatomic changes in the context of intestinal subset specialization. The most pronounced difference between INT1 and INT2-9 cells is the enrichment of stress-response genes in INT1. Based on the present findings, together with our previous work identifying INS-7 as an INT1-secreted signal (PMID: 39127676), we propose that INT1 cells are sentinel enteroendocrine cells that integrate information from the luminal environment and the metabolic state of intestinal cells.

**Reviewer #3 (Public review):**

Summary:

In this study, Liu and colleagues utilize TRAP-seq to profile the repertoire of actively translated mRNAs in different intestinal cell types (anterior INT1 vs. posterior INT2-9 cells) in *C. elegans*. A key goal of this study was to identify transcripts differentially expressed/translated between these intestinal cell subtypes in the context of animals being well fed or subjected to acute (30 minutes) or chronic (3 hours) starvation, followed by refeeding.

The authors identify a number of differentially expressed genes across all of the conditions tested. They then provide an initial survey of the landscape of translatome changes through Weighted Gene Network Correlation Analysis (WGNA), and some high-level functional surveys via Gene Ontology (GO) term analysis and protein domain analysis. The authors validate the enriched expression patterns of some of their identified candidate genes using fluorescent promoter fusion reporters, confirming INT1-specific expression. The authors further implicate the role of several other candidate genes in pathogen avoidance and in response to nutritional cues by knocking them down specifically in INT1 cells by RNAi. Finally, the authors identify pyruvate as a major nutrient signal coming from the bacterial diet that suppresses the release of a key insulin peptide (INS-7), and identify some of the genes expressed in INT1 that are required for this response.

Strengths:

(1) Good use of and justification for TRAP-seq, because scRNA-seq would be difficult under the varied conditions used (starvation, refeeding).

(2) The manuscript is generally clear to read, and the data are generally well-presented with good supporting data that includes replicates, sample sizes, error measurements, and associated statistics.

(3) The dataset will be an interesting resource to mine for future studies focusing on mechanisms of how particular intestinal cell types respond to different environmental signals.

Weaknesses:

(1) A limitation of TRAP-seq, although powerful, is that only relative comparisons can be made between genotypes/conditions to identify differentially-expressed genes, rather than assessing whether a given gene is expressed at a certain level in a cell type under a certain condition. This limitation is due to the non-specific association of sticky RNA species with the beads during the immunoprecipitation step. This is a minor point, however, and the authors do a nice job of focusing their analysis on differentially expressed transcripts in the current study.

We agree that a limitation of TRAP-seq is that it is best suited for relative comparisons across cell types or conditions, rather than for determining the absolute expression level of a given transcript in a specific cell type. As the reviewer notes, this limitation arises in part from nonspecific recovery of background or sticky RNAs during the immunoprecipitation step, complicating the interpretation of absolute expression levels. For this reason, our analysis was designed to focus primarily on differentially enriched transcripts between INT1 and INT2-9 cells and across feeding states, rather than on assigning absolute expression levels to individual genes. We appreciate the reviewer’s recognition of this point. Our study uses TRAP-seq specifically to define relative translatomic differences between intestinal subsets and physiological states, which is well aligned with the strengths of this approach.

(2) Another limitation of the current study is that the experiments testing the role of candidate genes identified by their profiling experiments do not delve a bit deeper into providing a mechanistic understanding of the phenotypes being studied. At present, the results are thus viewed more as a genomics-based screen with some limited follow-up on interesting hits. However, this reviewer appreciates that when placed in the context of the work presented, a presentation of the profiling data along with some validation is an excellent starting point for future mechanistic studies elaborating on these interesting candidates.

We agree that the current study does not fully resolve the molecular mechanisms by which the candidate genes identified by TRAP-seq regulate the phenotypes examined here. Our primary goal was to generate a spatially resolved translatomic framework for INT1 and INT2-9 cells across feeding states, and to perform focused validation of selected candidates to establish the physiological relevance of the profiling results. We therefore view the current functional analyses as an initial validation and proof of principle, rather than a comprehensive mechanistic dissection of the molecular pathways for each candidate. We appreciate the reviewer’s recognition that these findings provide an excellent starting point for future studies.

Appraisal of whether the authors achieved their aims, and whether the results support their conclusions:

The main goal of the study was to survey the dynamic responses at the level of actively translated mRNAs of the INT1 vs INT2-9 cells in response to metabolic challenge.

Overall, the authors use established methods to perform their genome-wide analysis, and the set of differentially regulated genes is enriched for expected molecular functions and forms coherent networks in anticipated pathways.

The validation experiments (promoter::GFP fusion reporters, INT1-specific knockdowns of highly regulated genes) further corroborate the quality of the TRAP-seq datasets generated.

I have a few points for the authors that would further strengthen this work:

(1) The authors rightfully focus on the top differentially-regulated candidates, but it's unclear at present how far down their fold change list would lead to expression pattern validations. It would be useful to test a few more promoter::GFP fusion reporters at different enrichment/fold-change/statistical cutoffs.

Testing additional promoter::mNeonGreen reporters across a wider range of fold-change and statistical thresholds could be somewhat useful for calibrating ranked TRAP-seq candidate genes. However, given the variation in strains bearing extrachromosomal arrays, we did not consider this a stringent enough test, given that the sensitivity and dynamic range of RNA-seq far outpaces genetic fluorescence-based reporters. For these reasons, we focused on the top differentially enriched candidates to provide not only an initial validation of the dataset, but also to determine whether these candidates regulate biological functions in INT1 cells and thus serve as potentially useful biological readouts in future efforts.

(2) Although the INT1-specific RNAi provides a convenient strategy for rapidly perturbing and testing genes of interest for phenotypes, independently validating the knockdowns with genetic mutants, or alternatively (if genes are essential), degron alleles.

We agree that validating the INT1-specific RNAi phenotypes with independent genetic approaches, including null-allele or degron-based alleles for essential genes, would further strengthen the conclusions. In the current study, we used INT1-specific RNAi as a rapid and spatially restricted strategy to functionally test candidates identified by TRAP-seq and to determine whether these genes contribute to the specialized physiological functions of INT1 cells. We consider these experiments an initial validation of candidate function rather than a complete genetic dissection, which could be conducted in future efforts to study other aspects of INT1 function.

Impact:

The TRAP-seq data and list of differentially-expressed candidate genes will form an interesting set of high-priority candidates to study for their role in the reception and transduction of nutritional cues in response to food status and pathogens. This data will thus benefit the *C. elegans* community of researchers studying the mechanisms governing these phenomena.

**Recommendations for the authors:**

**Reviewer #1 (Recommendations for the authors):**

Major comments:

(1) The authors need to describe the fasting method used in detail. Was fasting performed on unseeded NGM plates or in liquid (M9 buffer)? Were the animals washed with buffer prior to starvation? If yes, how many times? These details are critical for any researcher to follow up on their results.

We have clarified the fasting/refeeding procedure in the revised Methods section. Briefly, worms were washed off OP50-seeded NGM plates with M9 buffer, washed three times in M9 buffer, and then transferred to unseeded NGM plates for fasting. For refeeding, worms were collected from the unseeded NGM plates with M9 buffer and transferred back to OP50-seeded NGM plates.

(2) The authors claim that "INT1 and INT2-9 cells maintain fundamentally different molecular identities independent of any and all acute or chronic conditions", which they primarily based on Principal Component Analysis (PCA). The circles shown in Figure 2A are arbitrary, and many such circles can be drawn in the 2D space to separate the samples in different ways. The authors should show this comparison in a translatome-wide similarity heatmap with hierarchical clustering (similar to Fig.2C, but with all the experimental conditions and their replicates on both x- and y-axes).

The ellipses shown in Figure 2A were generated using the stat_ellipse() function in ggplot2, which calculates the mean and covariance of the PC1 and PC2 for each line and draws ellipses corresponding to the 95% confidence level. The separation between lines is primarily driven by PC2, which accounts for 14% of the variance in the translatomic dataset. Although this difference is less pronounced when considering the full translatome, samples from the same line nevertheless cluster together, supporting line-specific differences in translatomic profile.

(3) Figure 4 of the study shows 18 Venn diagrams for genes that are differentially expressed between INT1 and INT2-9 cell types in fed, fasted, and refed conditions. In the absence of any statistical comparisons, it is difficult to interpret whether the extents of overlap (higher or lower than expected) are significant. Ideally, P values for hypergeometric tests should be provided for the overlap regions.

We appreciate the reviewer’s suggestion. We explored the use of hypergeometric testing, implemented through the SuperExactTest package in R, to assess the statistical significance of the overlaps shown in the Venn diagrams. However, this analysis yielded significant P values for essentially all overlap regions, including cases in which the degree of overlap was not especially informative and did not align with the interpretation presented in the text. This outcome likely reflects the dependence of the test on the size of the input gene sets and background universe, which can make statistical significance difficult to interpret meaningfully in this context.

(4) The claims made in lines 242-244 (Figure 5D) need to be supported by P values from hypergeometric tests.

Similar to the previous point.

(5) The interpretation of Figures 6E and 6F described in lines 296-297 needs to be supported by statistical analyses. The authors claim that the undulating pattern of expression of the turquoise module genes is stronger in INT1 compared to INT2-9. However, based on Figures 2C and 6F, it appears that the expression change is not necessarily weaker in INT2-9, but instead is different, i.e., the expression of turquoise module genes goes up during fasting in INT1 and goes down after refeeding, while their expression goes up during fasting and stays up after refeeding in INT2-9 cells.

We appreciate the reviewer’s point and agree that the turquoise module shows dynamic regulation in both cell populations. The key difference is not the presence versus absence of an undulating pattern, but rather the magnitude of that change, which is greater in INT1. Because of the limited number of biological replicates in some conditions, particularly the fasting group, we interpreted these results cautiously and used a nonparametric approach to assess differences in average module expression between states. This analysis indicated that the turquoise module changes significantly in both lines, but with a larger effect size in INT1. We have included the corresponding statistical analysis and effect size in the revised manuscript.

(6) Since the INS-7 coelomocyte uptake assay was used extensively in this study, some representative microscopy images should be included to complement the quantification.

We have added a new Figure 6G showing representative images corresponding to the quantification presented in Figure 6H.

(7) The authors claim that INT1-specific fmo-2 RNAi results in reduced basal INS-7 secretion, but they do not have the direct statistical comparison for this. Were experiments shown in Figures 6G and 6K done on the same day?

In the original Figure 6K (now Figure 6L), the data are presented as the percentage of normalized INS-7::mCherry fluorescence intensity relative to fed animals treated with vector RNAi. A statistical comparison between fed animals treated with INT1-specific *fmo-2* RNAi and fed vector RNAi controls was performed and was significant. We have also clarified that the experiments shown in the original Figures 6G and 6I (now Figures 6H and 6J) were performed on the same day.

(8) It is not clear why blocking the mitochondrial breakdown of pyruvate (Figures 7E and 7F) does not mimic the fasted state in terms of increased INS-7 secretion from INT1 cells. Doesn't this contradict the proposed model in this study? Can the authors speculate why this is the case?

We do not interpret inhibition of pyruvate dehydrogenase or pyruvate carboxylase as equivalent to the fasted state. Rather, our model is that fasting induces INS-7 secretion by lowering intracellular pyruvate in INT1 cells. Under this framework, blocking mitochondrial pyruvate breakdown would be expected to reduce pyruvate utilization and thus maintain intracellular pyruvate, preventing the drop in pyruvate that normally occurs during fasting. This would explain why these manipulations suppress fasting-induced INS-7 secretion. To directly examine this possibility, we performed the experiment in Figure 7G, which tests whether maintaining pyruvate levels in INT1 cells during fasting is sufficient to suppress INS-7 secretion. The results are consistent with this interpretation and further support a model in which decreased intracellular pyruvate is a key determinant of fasting-induced INS-7 secretion.

Minor comments:

(1) Figures 2E and 2G are very similar and represent the same result in two different ways (unbiased vs guided comparison). One of these should be moved to the supplementary figures.

Although these figures show similar patterns, they were derived from two independent analytical approaches, WGCNA and differential expression analysis. We therefore interpret the concordance between these independent methods as strengthening the robustness of the association and increasing confidence in the biological relevance of the observed pattern.

(2) In Figures S1C, S1D, and S1E, a more significant P-value is shown with a smaller circle, and a less significant P-value is shown with a larger circle. This is confusing to the reader and should be inverted.

We appreciate the reviewer’s comment and have removed the original Figure S1C–E, replacing it with a more informative analysis. The genes used in the original panel were drawn from the top markers reported for intestinal clusters in Ghaddar et al. (PMID: 37352352). However, these markers were defined by comparison with all *C. elegans* cell types, rather than by comparisons among anterior, middle, and posterior intestinal populations, and are therefore not optimal for resolving differences between intestinal subregions. Our further examination of marker expression across the intestinal clusters in the Ghaddar et al. (PMID: 37352352). dataset confirmed this limitation. These results underscore the strength of our dataset for identifying genes that distinguish INT1 from INT2–9. We also note that the spatial identities of the intestinal clusters in Ghaddar et al. (PMID: 37352352) were not clearly established in the text or by spatial transcriptomic evidence, making it difficult to assign the annotated anterior, middle, and posterior clusters to specific intestinal cells. We have revised the manuscript accordingly and replaced the original figure panels.

(3) Line 131 mentions the comprehensive characterization of the translatomic differences between INT1 and INT2-9 cells under each acute and chronic condition. However, the paragraph only discusses the differences in the fed condition. This is confusing, and the authors should mention the comparison between these cell types under acute and chronic conditions in subsequent sections where it is described.

In this paragraph, we indeed discuss the ‘fed’ condition, but in subsequent sections we follow with details analyses of acute versus chronic, as well as regional differences across the intestine. We have clarified this in the opening sentence of the referenced paragraph.

(4) The Venn diagrams in Figure 4 look very similar, and it is hard to differentiate between how 4A is different from 4I, how 4B is different from 4J, etc. The authors should include the labels for 'acute' or 'chronic' above each Venn diagram to guide the reader through these panels.

We have added labels indicating the acute and chronic conditions to the left side of each Venn diagram in the revised Figure 4.

(5) It is not clear in the figure legends how Figure 5E is different from Figure S6A, and how Figure 5F is different from Figure S7A. This should be better described in the figure legends.

We have added a sentence to better describe this in the figure legend.

(6) Figure 6C: Survival parameters such as median lifespan, number of animals for each condition, etc., should be reported for the different conditions.

We have revised Figure 6C to indicate the number of animals analyzed in each condition, and the median survival for each group is now reported in the corresponding figure legend.

(7) The colors used for control RNAi and clec-160 RNAi are very similar in Fig.6C. Easily distinguishable colors should be used.

We have changed the colors as suggested.

(8) The INT1-specific RNAi strain should be first described in line 285.

We have added the description for the INT1-specific RNAi strain in line 285.

(9) Line 304: 'REF' should be replaced with the reference.

We have replaced the “REF” with the reference (PMID: 39127676)

(10) The P value for statistical comparison between the fed and 30 min refed states should be shown in Figures 6G, 6I, and 6K.

We have now included the *p* value for the comparison as suggested. Figures 6G, 6I, and 6K are now labeled as 6H, 6J, and 6L, respectively.

(11) In Figure 7, the authors should consider replacing the 'refed' label with 'recovery' because the pyruvate treatment was done in the absence of 'feeding' (= bacteria consumption).

We appreciate the reviewer’s point. However, we chose to retain the label “refed” in Figure 7 to maintain consistency across the set of conditions examined, including 2% glucose and OP50 supernatant, which likewise do not involve bacterial consumption despite not showing effect on the refeeding response of INS-7 secretion.

(12) The full form of DISN should be mentioned in the figure legend of Figure 7.

We have included the full form of D1SN in the figure legend of Figure 7A.

(13) Line 367: 'normalization' should be replaced with 'return to basal levels'. 'Normalization of INS-7 secretion' might also mean normalization of INS-7::mCherry signal to CLM::GFP signal.

We have revised the wording per the reviewer's suggestion.

(14) The methods section has a quantitative RT-PCR section, but it is not clear if RT-PCR data are reported in any of the figures. Also, no qPCR primers are listed in Table S3.

We have removed the quantitative RT-PCR part from the methods section.

**Reviewer #2 (Recommendations for the authors):**

(1) The authors describe that the RPL-22-3xHA constructs are not integrated, at the very end, in the section "Limitations of the data". An earlier mention of this caveat would have been useful. In addition, it would help if the authors could provide their defense (which I think is very valid) of using non-integrated strains in the results section, as they describe the experimental setup. Also, some details were missing, which left me wanting to know: Were there expression differences? How were they accounted for? Was expression normalized between these two constructs, and if so, how?

The RPL-22-3xHA construct was integrated into each line to ensure more consistent transgene expression across experiments. Because the INT2-9 construct is expressed in a larger number of cells than the INT1 construct, the INT2–9 samples yielded greater amounts of pulled-down nascent RNA, as reflected in the supplemental table and in the higher aligned RNA counts observed for the INT2-9 samples. To account for these differences, differential expression analysis was performed using DESeq2, which corrects for library size by estimating sample-specific size factors with the median-of-ratios method. Raw counts are then normalized using these size factors, thereby accounting for differences in sequencing depth and minimizing confounding effects due to variation in library size. Such differences are common in RNA-seq experiments, particularly when comparing samples derived from distinct input populations.

(2) The 'acute' and 'chronic' exposures are thought through and carefully defined. The question I do have is whether the 3-hour fasting can be considered chronic fasting, given how surprisingly fast the animals lose their fat content. Could these kinetics indicate that the 30-minute fasting is reflective of mechanisms during which senses change in food availability, whereas the 30 minutes represents acute fasting (with chronic fasting - meaning fasting, during which the animal activated alternative pathways - occurring later)? While this may appear to be pure semantics, it could influence how the authors interpret their results. One method to more objectively separate an 'acute' from a 'chronic' stage may be to conduct a time course of fat loss-does fat loss plateau after 3 hours? The timing when the rate of decrease levels off could be more indicative of the beginning of a chronic phase.

We appreciate this important point and agree that it should be more clearly discussed. We interpret acute fasting as a pre-fat-loss state, since it is 30 minutes off food and no difference in fat levels are detectable at this stage (Fig 1B). The translatomic changes observed under acute fasting therefore likely reflect food-sensing mechanisms and early preparatory responses that promote subsequent fat mobilization. In contrast, chronic fasting (180 minutes off food – see Fig 1D) appears to represent a post-fat-loss state, in which fat stores have already been depleted, and the corresponding translatomic changes likely reflect the effects of sustained metabolic stress.

(3) The age of the animals used has to be more explicitly stated. Were these animals egg-laying? Or L4/young adults? This is likely to impact the changes that the animals undergo.

Day 1 young adults were subjected to the fasting. Great care was taken to ensure consistency across biological replicates.

(4) What is the rationale, in the authors' view, that stress response genes are apparently more enriched than metabolic or mitochondrial enzymes, and membrane receptor changes? Are the latter mostly regulated by PTMs/localization changes, etc?

Based on our current data, we cannot exclude the possibility that metabolic or mitochondrial enzymes, as well as membrane receptors, are regulated in INT1 and INT2–9 cells through mechanisms not captured at the translatome level, including post-translational modification or changes in subcellular localization under different fasting and refeeding conditions.

(5) The refeeding experiment with latex beads and killed OP50 is very clever. Details on when INS-7 was evaluated in the caoelomocytes would help the reader better understand and interpret these results.

INS-7mCherry signal was evaluated in the coelomocytes immediately after refeeding; we included this information in the methods section and referenced our previous paper.

(6) In the Discussion, I was looking for a more detailed context for how to think about the differences and similarities in the RNA-seq data between the two segments, and perhaps a discussion of whether there were any indications that the two segments communicated with each other.

The data show that the most pronounced difference between INT1 and the rest of the intestine at the RNAseq level, is the expression of stress response genes in INT1. Although there are some nuanced differences, the prevalence of stress response genes persists across feeding and fasting conditions. This difference, combined with the evidence that INT1 cells secrete the enteroendocrine peptide INS-7 (Fig 6 and PMID: 39127676) is strongly reminiscent of the mammalian enteroendocrine cells, which also secrete peptides and show strong expression of stress response genes (PMID: 37626258 and 27148273). We suggest that this category term reflects not only a canonical stress response, but also a broader response to shifts in the luminal environment, which INT1 cells are anatomically poised to detect well before the absorption of nutrients has begun further down the intestine (INT2-9). Thus, we believe INT1 cells are a newly defined enteroendocrine cell type within the *C. elegans* intestine.

Regarding communication between INT1 and INT2-9 – this is an intriguing possibility that we have considered, given that peptide genes and receptors are found in the RNAseq datasets. The extent to which the expression of these genes leads to functional effects is the subject of future investigation.

**Reviewer #3 (Recommendations for the authors):**

(1) Figure 1A - It would be better to also show single fluorescent protein channels to assess the specificity of the expression patterns. A schematic or labels of where the INT1 vs. INT2-9 boundaries are located would be helpful to non-experts.

(2) Figure 4 - At present, the Venn Diagrams are a very complicated way to visualize all of the comparisons/conditions. I would recommend that the authors consider using UpSet plots to better summarize the relevant comparisons they would like to make. The same consideration applies to Figure 5D.

(3) Line 301 - Description of the INT1-specific RNAi strategy. I think it would be better to bring this information earlier, close to line 285, where the authors first mention performing INT1-specific RNAi experiments.

We have added the description for the INT1-specific RNAi strain in line 285.

(4) Line 304 - I think the authors meant to cite a reference where the REF placeholder text is found

We have replaced the “REF” with the reference.
